# Covariations between Shell-Growth Parameters and the Control of the Ranges of Variation of Functionally Relevant Shell-Shape Parameters in Bivalves: A Theoretical Approach

**DOI:** 10.1155/2014/326832

**Published:** 2014-11-18

**Authors:** Jean Béguinot

**Affiliations:** ^1^Société d'Histoire Naturelle du Creusot, 12 rue des Pyrénées, 71200 Le Creusot, France; ^2^Biogéosciences, Université de Bourgogne, 21000 Dijon, France

## Abstract

Major traits of shell shape in bivalves may alternatively be described in terms of (i) *functionally relevant* parameters, assumed to play a significant role in the adaptation of bivalves molluscs to their environments (such as the shell-outline elongation *E*, ventral convexity *K*, and dissymmetry *D*), or (ii) *growth-based* parameters, directly controlled by the animal. Due to the geometrical linkage between functionally-relevant and growth-based parameters, adaptive constraints that may either widen or narrow the respective ranges of variations of the functional parameters lead to the onset of specific covariations (either positive or negative) between the growth-based parameters. This has practical interest since adaptive constraints are often difficult to identify directly, while they can be conveniently inferred indirectly *via* the easily recorded patterns of covariances between growth-based parameters. Hereafter, I provide the theoretical background of this tool, including (1) establishing the geometrical relationships between growth-based and functionally relevant parameters and (2) then specifying the correspondences between the different patterns of adaptive constraints, widening or narrowing the variations of the functional parameters and the corresponding patterns of covariations between the growth-based parameters. Illustrative examples of the practical use of this tool are provided, considering both interspecific and intraspecific variations within marine and fresh-water clams.

## 1. Introduction

The shell shape in bivalves—in particular the elongation *E*, ventral convexity *K*, and dissymmetry *D* of the shell outline—arguably has significant functional implications regarding animal fitness (*E*, *K*: [1–21]; *D*: [22]). Accordingly, “*functionally relevant*” parameters describing major aspects of the shell outline such as *E*, *K*, and *D* are likely submitted to significant selective pressures.

At the intraspecific level, selection is expected to more or less restrict the ranges of individual variations of the functionally relevant parameters within acceptable limits, according to both the environmental variability and the tolerance capacity of individuals.

At the interspecific level, selection may either (i)* increase* the range of variations between species, so as to promote the exploitation of sufficiently separate niches by distinct species or, conversely, (ii) tend to* decrease* the range of variations between species, in spite of niches diversification, in order to remain in the vicinity of a common adaptive optimum. For example, in a series of clam's genera, the interspecific variability of the ventral convexity *K* of shell contour is shown to be severely restricted, contrasting with an increased range of interspecific variations of the shell elongation *E* (Section 5). In short, to maintain at best the animals' fitness, the magnitude of the range of variations of each functionally relevant parameter may be either expanded or reduced, depending, in particular, upon the environmental context, as discussed below.

Now, as important as the functionally relevant parameters *E*, *K*, and *D* may be in bivalves, the animal has no direct control upon them however; it is only an* indirect* influence,* via* the control of “*growth-related*” parameters. This is because the shape of shell outline is not a geometrical figuration generated* per se*, defined at the outset, but it is the cumulative result of an accretionary growth process [4]. The animal continuously controls the rate of peripheral accretion of new material at each location of the shell contour [4, 23]. And it is through the relationships linking the controlled growth-related parameters to the resulting functionally relevant parameters that the shell outline may actually be regulated—indirectly by the animal.

Thus, understanding how the animal indirectly controls the functionally relevant parameters of shell shape, such as *E*, *K*, and *D*, requires the prior derivation of the set of geometrically based equations linking growth-related parameters (*α*, *ρ*, and *δ*, defined later on) to the functionally relevant parameters *E*, *K*, and *D*.

Due to this geometrical linkage, it will be shown that any particular pattern of constraints—widening or narrowing the respective ranges of variation of each functionally relevant parameters *E*, *K*, and *D*—implies the onset of a corresponding particular pattern of covariations (positive or negative) between the growth-related parameters *α*, *ρ*, and *δ* (and* vice versa*).

In short, the rational behind this is as follows.

- If a given functionally relevant parameter has geometrical dependences of the* same* sign (either >0 or <0) upon two growth-based parameters, then a* positive covariance* between these two growth-based parameters will tend to* widen* the range of variations of the considered functionally relevant parameter (as compared to what would be this range of variations if there was no covariance between these two growth-based parameters) and* vice versa*. Conversely, a* negative covariance* between these two growth-based parameters will tend to* narrow* the range of variations of the considered functionally relevant parameter.
- If a given functionally relevant parameter has geometrical dependences of* opposite* signs upon two growth-based parameters, then a* positive covariance* between these two growth-based parameters will tend to* narrow* the range of variations of the considered functionally relevant parameter and* vice versa*. Conversely, a* negative covariance* between these two growth-based parameters will tend to* widen* the range of variations of the considered functionally relevant parameter.

Biunivocal correspondences may thus be established between (i) the six possible patterns of adaptive constraints, either widening or narrowing the respective ranges of variations of the three functional parameters *E*, *K*, and *D* and (ii) six corresponding patterns of covariations (either positive or negative) between the three growth-based parameters *α*, *ρ*, and *δ*.

In practice, this set of correspondences provides a convenient tool, allowing for inferring indirectly which particular pattern of adaptive constraints actually affects the respective magnitudes of variation of the three functional parameters, on the basis of the pattern of covariances actually recorded between the three growth-based parameters.

And this is of significant practical interest, since the occurrence of adaptive constraints applyed to functionally relevant parameters is often difficult to detect and identify directly, while the patterns of covariances between growth-based parameters are far more easily recorded.

Hereafter, the theoretical background of this tool is described.

(1) The set of biunivocal geometrical relationships between the growth-based and the functionally relevant parameters describing shell shape in bivalves is derived at first.
(2) Then, the six patterns of correspondences are settled (Table 2) between (i) the six different possible patterns of constraints either widening or narrowing the respective ranges of variations of the three functional parameters *E*, *K*, and *D* and (ii) the six related patterns of covariation (either positive or negative) between the three growth-based parameters *α*, *ρ*, and *δ*.

Illustrative examples of the practical use of this tool are provided, including both interspecific and intraspecific variations within both marine and fresh-water clams.

## 2. Methods

Two alternative sets of descriptive parameters of the shell outline (the* growth-related* parameters *α*, *ρ*, and *δ* and the* functionally relevant* parameters *E*, *K*, and *D*) are defined first and the corresponding set of equations linking *E*, *K*, and *D* to *α*, *ρ*, and *δ* is then derived. The nine partial derivatives of these equations {∂*E*/∂*α*, ∂*E*/∂*ρ*, ∂*E*/∂*δ*, ∂*K*/∂*α*, ∂*K*/∂*ρ*, ∂*K*/∂*δ*, ∂*D*/∂*α*, ∂*D*/∂*ρ*, ∂*D*/∂*δ*} provide the sign and strength of the dependences of each parameters *E*, *K*, and *D* with respect to all parameters *α*, *ρ*, and *δ*. In turn, this data is used to disclose the six different patterns of covariances that will occur between growth-related parameters *α*, *ρ*, and *δ*, according to the six different possible patterns of constraints that may either widen or narrow the respective ranges of variations of the three functional parameters *E*, *K*, and *D*.

**Figure 1:**
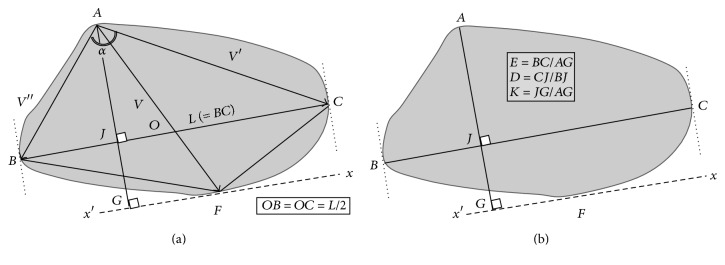
Definition of two alternative sets of descriptors of the shell outline. (a) The three growth-based parameters: apical angle $\alpha \;\;(=B\hat{A}C)$; differential growth index *ρ* = *V*/(1/2)(*V*′ + *V*′′); dissymmetric growth index *δ* = *V*′/*V*′′; (b) the three functionally relevant parameters: elongation *E*, dissymmetry *D*, and ventral convexity *K*.

In a* growth-based* approach of shell shape, the sagittal outline of shells may be appropriately parameterised synthetically using three indices associated with three “typical growth vectors” *V*, *V*′, and *V*′′ (Figure 1), each of them extending from the valve umbo *A*. The umbo (or “apex”) is defined, here, as* the extreme dorsal side near the umbo itself*, as quoted by Galtsoff [24]; see also [11]. Segment *BC* is the valve length *L*, and then vectors *V*′, *V*′′, respectively, join the apex *A* to the shell outline at the extremities *B*, *C* of the segment *BC* and vector *V* joins the apex *A* to the shell outline at point *F* via the middle *O* of segment *BC*. Finally, the segment *AG* is perpendicular to *xx*′, the parallel through *F* to *BC*.

Three* growth-based* indices are defined as

(i) the* apical angle* “*α*” (angle $B\hat{A}C$);
(ii) the* differential growth index* “*ρ*” identified to the ratio between axial (dorsoventral) growth and mean lateral growth, *ρ* = *V*/(1/2)(*V*′ + *V*′′);
(iii) the* dissymmetric growth index* “*δ*” identified to the ratio of the larger to the smaller lateral growth vectors, *δ* = *V*′/*V*′′.

These three parameters are* geometrically independent* factors, in the sense that no mutual dependence between *α*, *ρ*, and *δ* is compelled by purely geometric constraint: the direction and/or module of each vector may, indeed, freely be changed independently of the two others, in a purely geometric perspective.

These three parameters thus account schematically for the* growth pattern* of valves.

Alternatively, in a functionally relevant approach, the main traits of the shape of shell outline may be synthetically characterised by (i) the shell elongation, that is, the ratio of contour length to contour height, (ii) the valve dissymmetry, namely, the degree of dissymmetry of the position of the umbo versus the anterior and posterior extremities of shell, and (iii) the ventral convexity, that is, the degree of prominence of the ventral side of the shell outline, opposite to umbo. Three indices are defined correspondingly (Figure 1): the “shell elongation” index “*E*” as the ratio $BC/AG=L/(V\cdot \cos\;\;(G\hat{A}F))$, the “shell dissymmetry” index “*D*” as the ratio *CJ*/*BJ*, and the “ventral convexity” index “*K*” as the ratio *JG*/*AG*.

Note that choosing, in both approaches, a limited number of parameters to describe the shell outline, rather than implementing more refined approaches, such as Fourier analysis of shell contour, is deliberate. As the shell outline in bivalves is generally relatively simple, the main traits of shell outline may be fairly well captured by even a limited number of appropriately chosen parameters [25]. Moreover, a major advantage of limiting the number of parameters is that the equations linking growth-based and functionally relevant shape parameters may be derived under an explicitly* analytical* form, as such more appropriate to readily bring out and highlight the rational behind the equations.

As for the three growth-based parameters *α*, *ρ*, and *δ* above, these three functionally relevant parameters *E*, *D*, and *K* are, intrinsically, free from any geometrical constraints* a priori* and thus mutually independent. Yet, *E*, *D*, and *K* are entirely dependent* a posteriori* upon *α*, *ρ*, and *δ*, according to the three geometrically based equations below (see Appendix for further details and a demonstration):

(1) $$\begin{array}{c} E=f\left(\alpha ,\rho ,\delta \right),\\ D=g\left(\alpha ,\rho ,\delta \right),\\ K=h\left(\alpha ,\rho ,\delta \right). \end{array}$$

As focus is put here on intra- and interspecific variations, shell measurements were performed when the shells have reached the stage where their shape becomes substantially stabilized [26].

## 3. The Dependence of* Functionally Relevant* Parameters upon* Growth-Related* Parameters

The way each of the three functionally relevant parameters *E*, *D*, and *K* depends upon each of the three governing growth-related parameters *α*, *ρ*, and *δ* may be quantified by considering the corresponding partial derivatives ∂*E*/∂*α*, ∂*E*/∂*ρ*, ∂*E*/∂*δ*, ∂*D*/∂*α*, ∂*D*/∂*ρ*, ∂*D*/∂*δ*, ∂*K*/∂*α*, ∂*K*/∂*ρ*, ∂*K*/∂*δ*. In short (summarised at Table 1).

(i) The shell elongation *E* is (as expected) monotonously increasing with the apical angle *α* and monotonously decreasing with the differential-growth index; the dependence of *E* upon the growth-dissymmetry index *δ* is less intuitive but is also monotonously positive.
(ii) The shell dissymmetry *D* is strongly increasing with the growth-dissymmetry index *δ*, less intuitively decreasing with the apical angle *α* and strictly independent of the differential-growth index *ρ* and independent of the differential-growth index *ρ*.
(iii) The ventral convexity *K* of the shell outline is strongly increasing with both the apical angle *α* and the differential-growth index *ρ* and more weakly decreasing with the growth-dissymmetry index *δ*.

## 4. The Patterns of Covariances between Growth Parameters and the Corresponding Patterns of Constraints upon the Magnitude of Variation of Functional Parameters

As mentioned in Section 1, the covariances between growth-related parameters *α*, *ρ*, and *δ* are directly influential upon the respective magnitudes of the ranges of variations of the functionally relevant parameters *E*, *D*, and *K*, according to the sign of the dependence of each parameter *E*, *D*, and *K* upon each parameter *α*, *ρ*, and *δ* (signs of dependence provided at Table 1). From a theoretical point of view, six types of covariance may* a priori* possibly occur between couples of parameters among *α*, *ρ*, and *δ*. These types of covariance are listed at Table 2 with their corresponding influence upon the magnitudes of variations of *E*, *K*, and *D*, respectively (as compared to what these ranges would be in case of mutual independence between *α*, *ρ*, and *δ*).

For example, a positive covariance between *ρ* and *δ* (i) would correspond to a reduction of the ranges of variations Δ*E*, Δ*K* of both *E* and *K*, because both these parameters have dependences of opposite signs upon *ρ* and *δ* (see Table 1) and (ii) would not affect the range of variations of *D* since *D* is independent of *ρ*. Similarly, a negative covariance between *α* and *δ* would correspond (i) to increased range of variations Δ*K*, Δ*D* for both *K* and *D*, as the latter both have dependences of opposite signs upon *α* and *δ* (see Table 1) and (ii) to a reduced range of variations Δ*E* of *E*, since *E* has dependences of the same sign upon *α* and *δ*.

The linkage is thus highlighted between the (presumably selection-induced) patterns of constraints applying to the respective magnitudes of variation of the different functionally-relevant parameters of shell shape and the corresponding patterns of covariance between the growth-based parameters, placed under the animal control.

Interestingly, this linkage, between the patterns of variability of shell* shape* and specific patterns of covariances between shell growth parameters, is mirrored in both gastropods [27, 28] and bivalves [29] by a rather similar kind of linkage between the degree of variability of shell-*size* and (once again) a specific type of covariance between shell growth parameters.

## 5. The Model Compared to Field Data

As already pointed out (Section 2), the three growth-related parameters *α*, *ρ*, and *δ* are, fundamentally,* geometrically independent* factors. Accordingly, the occurrence of covariances between these parameters is not expected* a priori*. And if any covariance, nevertheless, is observed, it should then find its origin out of pure geometry, in some kind of biological constraint. In turn, such a biological constraint might have either (i) a* developmental* origin, thus applying directly to *α*, *ρ*, *δ*, or (ii), an* adaptive* origin, directly applying to any of *E*, *K*, and *D* and, consequently affecting only indirectly, *α*, *ρ*, and *δ* (through the relationships (1)).

The possible occurrence of covariances between growth-related parameters *α*, *ρ*, and *δ* was thus investigated in a series of cases (Béguinot,* unpublished results*):

- at the interspecific level, within the major genera belonging to the super-family of marine bivalves* Tellinidea* (Blainville 1814):* Tellina* Linnaeus 1758,* Donax* Linnaeus 1758,* Gari* Schumacher 1817,* Abra* Leach* in* Lamarck 1818,* Macoma* Leach 1819;
- at the intraspecific level, within a common marine species,* Donax trunculus* and two fresh-water bivalves,* Unio pictorum* (Linnaeus 1758), and* Anodonta cygnea* (Linnaeus 1758).

Two distinct types of covariances occur, depending on whether interspecific variations or intraspecific variations are considered (Table 3).

Within each of the five genera examined, the interspecific variations of the apical angle *α* and of the differential-growth index *ρ* were systematically* negatively correlated*, with the trend being highly significant. No significant covariance was recorded between *α* and *δ* nor between *ρ* and *δ*. Now, for each of the three species examined, the intraspecific variations of the differential-growth index *ρ* and of the growth-dissymmetry index *δ* were systematically* positively correlated*, with the trend being highly significant. No significant covariance was recorded between *α* and *ρ* nor between *α* and *δ*.

**Figure 2:**
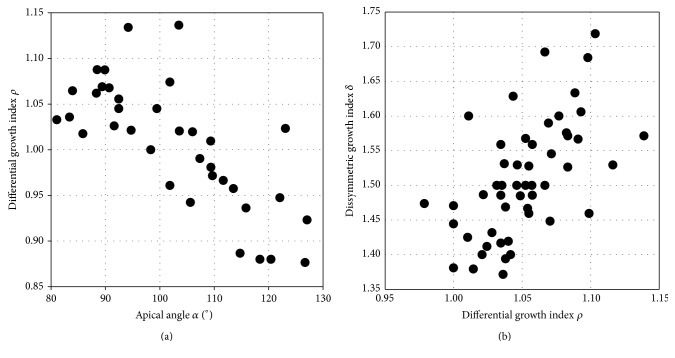
Statistically significant covariances between growth-based parameters. (a) Interspecific variations within the genus* Donax* (36 species): a negative covariance between *α* and *ρ* (*r* = −0.74, *P* < 0.0001; no covariance between *ρ* and *δ*: *r* = 0.04, *P* > 0.41). (b) Intraspecific variations within the* Donax trunculus* (51 individuals): a positive covariance between *ρ* and *δ* (*r* = +0.58, *P* < 0.0001; no covariance between *α* and *ρ*: *r* = 0.04, *P* > 0.39).

Figures 2(a) and 2(b) illustrate graphically the recorded covariances between growth-related parameters, for coquina clams* Donax*.

## 6. Discussion

The results above show that, in spite of their geometrical independence* a priori*, growth-related parameters *α*, *ρ*, and *δ* may actually be strongly covariant, in both intra- and interspecific contexts. These covariances must therefore rely on some source of biological constraints since a purely geometrical origin is excluded. Distinguishing between the two main types of biological constraints that may be considered here—developmental or adaptive—remains, however, far from being easy [30]. A few remarks, however, may provide suggestive clues.

Interestingly, for each of the three cases involving intraspecific variations (*Donax trunculus, Unio pictorum*, and* Anodonta cygnea*), the recorded type of covariance (covar. *ρ* − *δ* positive) is the only one, among the six, which leads globally to the narrowest ranges of variations of each of the functionally relevant descriptors of shell-shape (Table 2) and this is precisely what could be expected for intraspecific variations, as mentioned above. Now, for each of the five cases involving interspecific variations (*Tellina, Donax, Gari, Abra, and Macoma*), the recorded type of covariance is consistently different (covar. *α* − *ρ* < 0) and favors the enlargement of the range of variations of the shell elongation *E* (Table 2), therefore promoting, as expected, functional differentiation between species within the same genus, in this respect. In short, in both cases, these results actually make sense according to the same perspective: reducing the range of variations of a functionally relevant phenotypic character (the shell elongation *E*) at the intraspecific level and, on the contrary, contributing to enlarging this range at the interspecific level. The range of variations of the ventral convexity *K*, for its own, constantly remains narrow, at the intraspecific level and at the interspecific level as well, within all the six genera investigated. Presumably, some significant constraint specifically opposes any excessive variation of this particular trait of shell shape. Some tentative arguments may be suggested, regarding the selective advantage that might be associated with a limited degree of variability of the ventral convexity, even at the interspecific level. For example, one may note that the ventral portion of valves is often considered as the weakest part and, thus, more at risks [3], since the ventral part of the shell, especially the posteroventral sector, is ordinarily less thick and thus less resistant than the dorsal part. Increasing ventral convexity, that is, ventral prominence, would thus enlarge the corresponding weakened zone. Also, a larger convexity would tend to reduce the sealing pressure along the ventral margin (at given unchanged positions of insertion of the adductor muscles) and thus would make easier the shell opening by predators. Accordingly, a sufficient level of shell mechanical resistance to various kinds of environmentally induced stresses might preclude too high values of ventral convexity. Conversely, a sufficient value of ventral convexity might well be dictated by the avoidance of excessively acute profiles of valves contour at the anterior and posterior extremities (i.e., around *C* and *B*, Figure 1) which would inevitably result from a too weak convexity. Such acute portions would be at still greater risks and more prone to suffering local breakage. An optimally centred and size-limited range of values for the ventral convexity of shell outline would thus arguably be selected.

Overall, the above remarks seem pretty much in agreement with the hypothesis of adaptive selection as the likely cause responsible for the constraints governing the respective magnitudes of variation of the functionally relevant parameters *E*, *K*, *D*. According to this hypothesis, the recorded covariances between growth-related parameters would be the* indirect* byproduct of the selective processes* directly* governing the respective magnitudes of variations (either intra- or interspecific) of the functionally relevant parameters.

Yet, many questions remain open to investigation: (i) is the positive *ρ* − *δ* covariance an exclusive feature of intraspecific variability? (ii) beyond the evidences reported here, are covariances between growth-related parameters a general trait among bivalves? (iii) besides the two recorded type of covariances (positive covariance *ρ* − *δ* and negative covariance *α* − *ρ*) does other one(s) among the other four potential types (Table 2) actually occur in other families of bivalves? We hope the theoretical framework provided here may invite further investigations on these issues.

## Figures and Tables

**Table 1:** The signs of the variations, ∂*E*/∂*α*, ∂*E*/∂*ρ*, ∂*E*/∂*δ*, ∂*D*/∂*α*, ∂*D*/∂*δ*, ∂*K*/∂*α*, ∂*K*/∂*ρ*, and ∂*K*/∂*δ*, of the functionally relevant parameters, *E*, *K*, and *D*, according to variations of the growth-based parameters *α*, *ρ*, and *δ* (according to Section 2).

	∂*E*	∂*D*	∂*K*
/∂*α*	>0	<0	>0
/∂*ρ*	<0	=0	>0
/∂*δ*	>0	>0	<0

**Table 2:** Consequences on the magnitude of variations Δ*E*, Δ*K*, and Δ*D* of the functionally relevant parameters *E*, *K*, and *D*, according to the type of covariation between growth-related parameters *α*, *ρ*, and *δ*. Arrows pointing upward (resp., downward) stand for widened (resp., narrowed) ranges of variations; the sign “=” stands for a nonaffected range of variations [as compared to what would be these ranges in case of mutual independence between *α*, *ρ*, and *δ*].

Pattern of covariation of shell-growth parameters	Δ*E*	Δ*K*	Δ*D*
Covariance *ρ* − *δ* positive	↓	↓	=
Covariance *ρ* − *δ* negative	↑	↑	=
Covariance *α* − *ρ* positive	↓	↑	=
Covariance *α* − *ρ* negative	↑	↓	=
Covariance *α* − *δ* positive	↑	↓	↓
Covariance *α* − *δ* negative	↓	↑	↑

**Table 3:** Covariances between growth-related parameters *α*, *ρ*, and *δ*, in (i) *inter*-specific context (negative covariance *α* − *ρ* within genus *Tellina, Donax, Gari, Abra, and Macoma*) and (ii) *intra*-specific context (positive covariance *ρ* − *δ* within *Donax trunculus, Unio pictorum, and Anodonta cygnea*).

	*Tellina *	*Donax *	*Gari *	*Abra *	*Macoma *	*Donax tr. *	*Unio pic. *	*Anodont. *
Context	*inter*-sp.	*inter*-sp.	*inter*-sp.	*inter*-sp.	*inter*-sp.	*intra*-sp.	*intra*-sp.	*intra*-sp.
Covariance	*α* − *ρ* < 0	*α* − *ρ* < 0	*α* − *ρ* < 0	*α* − *ρ* < 0	*α* − *ρ* < 0	*ρ* − *δ* > 0	*ρ* − *δ* > 0	*ρ* − *δ* > 0
Correl. coeff.	−0.91	−0.74	−0.87	−0.94	−0.79	+0.58	+0.71	+0.81
Sample size	*n* = 49	*n* = 36	*n* = 11	*n* = 7	*n* = 7	51	121	57
Significance	*P* < 0.0001	*P* < 0.0001	*P* < 0.001	*P* = 0.002	*P* = 0.04	*P* < 0.0001	*P* < 0.0001	*P* < 0.0001

## Appendix

### The Equations Relating the Valve Shape Parameters *E*, *D*, and *K* to the Growth Parameters *α*, *ρ*, and *δ*

(∗)

(∗∗)

(∗∗∗)

with *X* = 2*δ*/(*ρ* · (*δ* + 1)); cos⁡(*θ*) = 2*δ* · sin(*α*)/(4*δ* ^2^sin^2^(*α*) + (*δ* ^2^ − 1)^2^)^0.5^ and *E* defined above.

NB: for the specific case where shell is (sub-)symmetric (*δ* = 1), the three equations are simplified as

(A.1) $$\begin{array}{c} E=\frac{{\left[2\left(1-\cos\left(\alpha \right)\right)\right]}^{0.5}}{\rho };\\ D=1;\\ K=1-\frac{{\left[\left(1/2\right)\left(1+\cos\left(\alpha \right)\right)\right]}^{0.5}}{\rho }. \end{array}$$

In other words, this system of three equations (∗), (∗∗), and ∗∗∗ expresses the tensor relationship linking the two alternative sets of parameters describing the shell-outline, *α*, *ρ*, *δ* and *E*, *D*, *K*.

*Demonstration of Equations* (∗), (∗∗),* and* ∗∗∗. The following, classical relationships between angles, sides, and height in triangles are applied here within the triangle *ABC* (see Figure 1):

(A.2) $$\begin{array}{c} BC=L={\left(V^{\prime 2}+V^{\prime \prime 2}-2V^{\prime }V^{\prime \prime }\cos\left(\alpha \right)\right)}^{0.5}, \end{array}$$

(A.3) $$\begin{array}{c} JC=\frac{\left(L^{2}+V^{\prime 2}-V^{\prime \prime 2}\right)}{\left(2L\right)}, \end{array}$$

(A.4) $$\begin{array}{c} AJ={\left[V^{\prime 2}-{\left(\frac{\left(L^{2}+V^{\prime 2}-V^{\prime \prime 2}\right)}{\left(2L\right)}\right)}^{2}\right]}^{0.5}. \end{array}$$

*(i) Valve Elongation. E* = *BC*/*AG* = *L*/*AG* = *L*/(*AF* · cos⁡(*θ*)) = *L*/(*V* · cos⁡(*θ*)), with *θ* = angle $G\hat{A}F$.

Accounting for the definitions of *ρ* = *V*/(1/2)(*V*′ + *V*′′) and *δ* = *V*′/*V*′′, consider the following:

(A.5) $$\begin{array}{c} V^{\prime \prime }=\frac{2V}{\left(\rho \cdot \left(\delta +1\right)\right)}. \end{array}$$

Equation (A.2) yields then

(A.6) $$\begin{array}{c} \frac{L}{V^{\prime \prime }}={\left(\delta^{2}-2\delta \cdot \cos\left(\alpha \right)+1\right)}^{0.5}. \end{array}$$

From (A.5) and (A.6),

(A.7) $$\begin{array}{c} \left(\frac{L}{V}\right)=\frac{2{\left(\delta^{2}-2\delta \cdot \cos\left(\alpha \right)+1\right)}^{0.5}}{\left(\rho \cdot \left(\delta +1\right)\right)}. \end{array}$$

Now, *JO* = *JC* − *L*/2 and from (A.3) and (A.4), consider the following:

(A.8) $$\begin{array}{c} tg\left(\theta \right)=\frac{JO}{AJ}=\frac{\left(V^{\prime 2}-V^{\prime \prime 2}\right)}{{\left(4L^{2}\cdot V^{\prime 2}-{\left(L^{2}+V^{\prime 2}-V^{\prime \prime 2}\right)}^{2}\right)}^{0.5}}, \end{array}$$

and as *δ* = *V*′/*V*′′,

(A.9) $$\begin{array}{c} tg\left(\theta \right)=\frac{\left(\delta^{2}-1\right)}{{\left(4\delta^{2}{\left(L/V^{\prime \prime }\right)}^{2}-{\left({\left(L/V^{\prime \prime }\right)}^{2}+\delta^{2}-1\right)}^{2}\right)}^{0.5}}. \end{array}$$

Substituting *L*/*V*′′ by its expression in (A.6) yields *tg*(*θ*) = (*δ*²−1)/(2*δ* · sin(*α*)) and then

(A.10) cos⁡θ=11+tg2θ0.5=2δ·sinα4δ2sin2α+δ2−120.5.

Finally, (A.7) and (A.10) yield, for the elongation *E* = *L*/(*V* · cos⁡(*θ*)),

(A.11) $$\begin{array}{c} E=\frac{{\left[\left(\delta^{2}-2\delta \cdot \cos\left(\alpha \right)+1\right)\cdot \left(4\delta^{2}\sin^{2}\left(\alpha \right)+{\left(\delta^{2}-1\right)}^{2}\right)\right]}^{0.5}}{\left(\rho .\left(\delta +1\right)\cdot \delta \cdot \sin\left(\alpha \right)\right)}. \end{array}$$

*(ii) Valve Dissymmetry. D* = *JC*/*JB* = *JC*/(*L* − *JC*) and, according to (A.2) and (A.3), *D* = (*δ*²−1 + (*L*/*V*′′)²)/(1 − *δ*²+(*L*/*V*′′)²). With (*L*/*V*′′) defined at equation (A.6), consider the following:

(A.12) $$\begin{array}{c} D=\frac{\left(\delta^{2}-\delta \cdot \cos\left(\alpha \right)\right)}{\left(1-\delta \cdot \cos\left(\alpha \right)\right)}. \end{array}$$

*(iii) Convexity K of the Ventral Contour of Valve.* The convexity *K* of the ventral contour of valve is defined by the ratio *K* = *JG*/*AG* = 1 − *AJ*/*AG*.

From equation (A.4) and accounting for *E* = *L*/*AG*, consider the following:

(A.13) $$\begin{array}{c} K=1-{\left[V^{\prime 2}-{\left(\frac{\left(L^{2}+V^{\prime 2}-V^{\prime \prime 2}\right)}{\left(2L\right)}\right)}^{2}\right]}^{0.5}\left(\frac{E}{L}\right)\\ K=1-E\cdot {\left\{{\left(\frac{V^{\prime }}{L}\right)}^{2}-0.25{\left[1+{\left(\frac{V^{\prime }}{L}\right)}^{2}-{\left(\frac{V^{\prime \prime }}{L}\right)}^{2}\right]}^{2}\right\}}^{0.5}. \end{array}$$

According to equation (A.5), *V*′′/*L* = 2/(*E* · *ρ* · (*δ* + 1)) and *V*′/*L* = 2*δ*/(*E* · *ρ* · (*δ* + 1)).

Substitution of *V*′/*L* and *V*′′/*L* by their expressions above yields finally

(A.14) K=1−E ·1+XE·cos⁡θ2−Xδ·E·cos⁡θ22XE·cos⁡θ2−0.25hhh×1+XE·cos⁡θ2−Xδ·E·cos⁡θ220,5

with *X* = 2*δ*/(*ρ* · (*δ* + 1)); cos⁡(*θ*) = 2*δ* · sin(*α*)/(4*δ* ^2^sin^2^(*α*) + (*δ* ^2^ − 1)^2^)^0.5^ according to (A.10) and *E* defined by (A.11).
